# *Massilia varians* P2-4 Supplementation Enhances Immunity, Antioxidant Capability, Intestinal Microbiota Diversity, and Disease Resistance Against *Pseudomonas aeruginosa* Infection in Chinese Mitten Crab *Eriocheir sinensis*

**DOI:** 10.3390/biology15120908

**Published:** 2026-06-10

**Authors:** Yiyao Liu, Yueqi Yang, Xurui Zheng, Haipeng Cao, Chunlei Gai, Weidong Ye

**Affiliations:** 1 National Pathogen Collection Center for Aquatic Animals, Shanghai Engineering Research Center of Aquaculture, Shanghai Ocean University, Shanghai 201306, China; 2 Marine Science Research Institute of Shandong Province (National Oceanographic Center, Qingdao), Qingdao 266104, China; chunlei317@163.com; 3 Department of Clinical Laboratory, Quzhou Affiliated Hospital of Wenzhou Medical University, Quzhou 324000, China

**Keywords:** *Massilia varians*, *Eriocheir sinensis*, protective effect, *Pseudomonas aeruginosa*

## Abstract

*Massilia varians* has potential as a probiotic to inhibit bacterial pathogens in aquaculture, but very limited information is available regarding its use in Chinese mitten crab *Eriocheir sinensis* for enhancing host immunity, antioxidant ability and intestinal microbiota homeostasis. In this study, a 40-day feeding trial was conducted to evaluate the protective effects of dietary supplementation with *M. varians* P2-4 on nonspecific immune response, antioxidant status, intestinal microbiota and resistance against *Pseudomonas aeruginosa* infection in *E. sinensis*. Results demonstrated that dietary supplementation with *M. varians* P2-4 at 6.0 × 10^6^ to 6.0 × 10^8^ CFU/g diet significantly boosted nonspecific immunity and improved antioxidant capability of *E. sinensis*, mainly as evidenced by markedly increased activities of plasma lysozyme, plasma superoxide dismutase, hepatopancreatic superoxide dismutase and catalase. Furthermore, crabs fed *M. varians* P2-4-supplemented diets exhibited markedly improvements in intestinal microbiota composition and diversity, and showed substantially enhanced survival following *P. aeruginosa* challenge, with 7-day relative percentage survival ranging from 76.9% to 100.0%. To the best of our knowledge, this is the first study to reveal that *M. varians* P2-4 supplementation functions as a new biocontrol strategy in *E. sinensis* by effectively improving the non-specific immunity, antioxidant status and intestinal microbiota to mitigate *P. aeruginosa* infection.

## Simple Summary

*Pseudomonas aeruginosa* is a clinically significant bacterial pathogen that poses a serious threat to the health and aquaculture sustainability of *Eriocheir sinensis*. However, there is currently limited information regarding the use of *Massilia varians* to confer *E. sinensis* against *P. aeruginosa* infection. Here, we performed a 40-day feeding trial to evaluate the effects of dietary supplementation with *M. varians* P2-4 on immunity, antioxidant capability, intestinal microbiota, and resistance to *P. aeruginosa* infection in *E. sinensis*. Comprehensive analyses revealed that *M. varians* P2-4 supplementation could improve immune and antioxidant responses, modulate intestinal microbiota, and enhance resistance to *P. aeruginosa* infection in *E. sinensis*. Our data elucidate *M. varians* P2-4 supplementation as a biocontrol strategy to confer effective protection in *E. sinensis* against *P. aeruginosa* infection.

## 1. Introduction

The Chinese mitten crab *Eriocheir sinensis* is an economically vital freshwater aquaculture species in China and other East Asian countries [1], and now represents the dominant contributor to global crab production owing to its high market value and consumer demand [2]. Especially in China, rapid advancements in intensive aquaculture technologies have elevated annual production of this crab to over 888,000 tons [3]. However, bacterial infections have become one of the major bottlenecks in sustainable *E. sinensis* aquaculture. For example, *Pseudomonas aeruginosa* infection has emerged as a critical threat to *E. sinensis* farming, triggering serious diseases such as hepatopancreatic necrosis disease and resulting in a mortality of over 40% [4]. Consequently, there is an urgent need to develop sustainable preventive strategies against *P. aeruginosa* infection in this species.

The genus *Massilia* belongs to the family *Oxalobacteraceae* within the class *Betaproteobacteria* [5]. Although a few *Massilia* isolates are occasionally associated with human septicemia and osteomyelitis [6], members of *Massilia* are increasingly recognized as a promising probiotic candidate [7] and exhibit great potential for the biocontrol of bacterial pathogens [8,9]. For instance, *M. varians* P2-4, an avirulent predatory bacterium possessing bacteriolytic ability and showing no virulence genes and non-pathogenicity to *E. sinensis* [8,10], exhibits potent antibacterial activity against pathogenic *Aeromonas hydrophila*, *A. caviae*, *Photobacterium damselae*, and *Shewanella algae* in aquaculture [8]. However, scarce information is available concerning the modulatory effects of probiotic *Massilia* on immunity, antioxidant status, intestinal microbiota and disease resistance in *E. sinensis*.

In the present study, a 40-day feeding trial was conducted in *E. sinensis* to evaluate whether dietary supplementation with *M. varians* P2-4 could improve immune and antioxidant responses, modulate intestinal microbiota, and enhance resistance to *P. aeruginosa* infection in *E. sinensis*. To our knowledge, this is the first study to reveal the beneficial effects of *M. varians* supplementation on innate immunity, antioxidant capability, intestinal microbiota, and resistance to *P. aeruginosa* infection in *E. sinensis*.

## 2. Materials and Methods

### 2.1. Animal Ethics

All the experimental procedures in this study were under the ethics committee of Shanghai Ocean University (No. SHOU-DW-2023-030).

### 2.2. Probiotic Strain

*M. varians* strain P2-4, a predatory bacterium previously isolated from aquaculture pond sediment and identified using phenotypical and molecular methods [8], was maintained at 30 °C on nutrient agar (NA; Sinopharm Chemical Reagent Co., Ltd., Shanghai, China) slants, and used in this study.

### 2.3. Experimental Diet Preparation

Prior to experimental diet preparation, *M. varians* P2-4 suspension was prepared following the protocol described by Huang et al. [11]. Specifically, *M. varians* P2-4 was inoculated into nutrient broth (NB; Sinopharm Chemical Reagent Co., Ltd., Shanghai, China), and incubated at 30 °C for 24 h with shaking at 180 r/min. *M. varians* P2-4 cells were harvested by centrifugation at 4000 r/min for 10 min, washed with sterile distilled water, and suspended in sterile distilled water to yield a uniform cell suspension. The suspension was quantified through counting colony forming units (CFU) on NA plates from a series of 10-fold dilutions in sterile distilled water [12]. The resulting suspension was diluted with sterile distilled water, and the dilutions were then uniformly sprayed onto a sterile commercial basal diet (Suqian Hongxiang Feed Co., Ltd., Suqian, China), whose formulation and proximate composition were previously reported by Cao et al. [13]. The mixtures were air-dried for 16 h under aseptic conditions at 25 °C until the suspension was fully absorbed by the diets. The viable cell counts in the diets were checked immediately after diet preparation, and quantified as 6.0 × 10^6^, 6.0 × 10^7^ and 6.0 × 10^8^ CFU/g diet based on the CFU enumeration on NA plates following a 10-fold serial dilution in sterile distilled water. The basal diet, subjected to the same handling procedure as the supplemented diets, was used as the control. All the experimental diets were prepared regularly at weekly intervals according to Kumar et al. [14], and were stored at 4 °C.

### 2.4. Crab Culture

Juvenile Chinese mitten crabs (17.51 ± 1.28 g in weight) were sourced from a crab farm in Nantong, Jiangsu, China, and acclimatized to laboratory conditions for 14 days, following the health status assessment through sampling 10 crabs for a careful examination of external appearance, physical behavior and absence of parasites, viruses and bacterial pathogens according to Feng et al. [15], Yang et al. [16], and Ding et al. [17]. Briefly, following a check of limb intactness, strong viability, and high shell hardness, a squash of organs (gill, hepatopancreas, and muscle) were made and examined as wet mounts for parasites under the microscope (YS100, Nikon, Tokyo, Japan). Simultaneously, the hepatopancreas was homogenized in sterile normal saline (1:1, *w*/*v*) for 15 min, and the resulting homogenate was centrifuged at 10,000 r/min for 20 min at 4 °C, and the supernatant was filtered through 0.22 µm-pore-size membrane filter, and the filtrate was dropped onto a formvar-coated copper grid, negatively stained with 2% sodium phosphotungstate, and observed under a transmission electron microscope (HT770, Hitachi, Tokyo, Japan) [17]. In addition, the hepatopancreas was directly streaked onto NA plates, and observed for the presence of bacteria following 24 h of incubation at 28 °C [18]. The crabs were distributed to 12 replicate aquaria (78 cm × 52 cm × 48 cm) with 120 L aerated tap water at pH 7.0–8.0, dissolved oxygen ≥ 6.0 mg/L, total ammonia ≤ 0.2 mg/L and 28 °C. Each aquarium as an experimental unit was equipped with three tiles placed at the bottom as shelters to avoid cannibalism [19]. Three replicate treatment aquaria (*n* = 50 crabs per aquarium as recommended by Dai et al. [20]) received experimental diets supplied with 6.0 × 10^6^, 6.0 × 10^7^ and 6.0 × 10^8^ CFU/g diet of *M. varians* P2-4, respectively, and three additional replicate aquaria (*n* = 50 crabs per aquarium) served as the control group and were fed the sterile basal diet under identical experimental conditions. All diets were offered twice daily (8:00 and 18:00) at a fixed feeding rate of 3% of the total body weight to apparent satiation for 40 days [21]. Experimental conditions were standardized by maintaining a 12 h dark: 12 h light photoperiod [22]. Uneaten food was siphoned from aquaria 2 h post-prandially, 1/3 of the aquarium water was replaced daily with fresh aerated tap water to support consistent growth and survival [4].

### 2.5. Plasma Immunity and Antioxidant Capacity Assay

Immediately following the feeding trial, haemolymph was collected from the base of third pleopods of five crabs from each aquarium in both control and treatment groups, using ice-chilled acid citrate dextrose (ACD) anticoagulant tubes [13]. The haemolymph of these five individuals from each aquarium were pooled into a single sample. The pooled plasma was subsequently separated by centrifugation at 4000 r/min for 20 min at 4 °C. Thereafter, the plasma acid phosphatase (ACP; catalog number A060-2-2), alkaline phosphatase (AKP; catalog number A059-2-2), lysozyme (LZM; catalog number A050-1-1), superoxide dismutase (SOD; catalog number A001-2-2), and catalase (CAT; catalog number A007-1-1) activities were quantified using commercial assay kits (Nanjing Jiancheng Bioengineering Institute, Nanjing, China) as recommended by Xu et al. [23], strictly adhering to the manufacturer’s instructions.

### 2.6. Hepatopancreatic Immunity and Antioxidant Capability Assay

Immediately post the feeding trial, the hepatopancreas tissues were rapidly dissected from five crabs from each aquarium in both control and treatment groups, and pooled into a single sample, which was kept on ice throughout the sampling process and stored at −80 °C for further analysis. The time interval from tissue dissection to final storage was strictly controlled within 30 min [24]. The pooled hepatopancreas sample was homogenized with sterile normal saline (1:9, *w*/*v*) for 15 min at 4 °C [23] using a homogenizer (Shanghai Jingxin Industrial Development Co., Ltd., Shanghai, China). The resulting homogenate was centrifuged at 2500 r/min for 10 min at 4 °C, and the supernatant was collected for determination of hepatopancreatic ACP, AKP, SOD, and CAT activities according to the protocol of commercial assay kits (Nanjing Jiancheng Bioengineering Institute, Nanjing, China) as recommended by Xu et al. [23].

### 2.7. Intestinal Microbiota Assay

Following the feeding trial, the intestines were aseptically excised from five randomly selected crabs in each aquarium across all control and treatment groups. Intestinal contents of these five individuals from each aquarium were then carefully collected following the method described by Hou et al. [25], and pooled into a single sample. Total genomic DNA was extracted from the pooled intestinal content samples using the DNA extraction kits (Tiangen Biotech. (Beijing) Co., Ltd., Beijing, China) following the manufacturer’s instruction. DNA integrity and approximate concentration were preliminarily assessed by 1% agarose gel electrophoresis. Subsequent quantification was performed using a NanoDrop 2000 UV-Vis spectrophotometer (Thermo Scientific, Wilmington, NC, USA). The V3–V4 hypervariable region of the bacterial 16S rRNA gene was amplified using primers (forward primers: 5′-ACTCCTACGGGAGGCAGCAG-3′, reverse primers: 5′-GGACTACHVGGGTWTCTAAT-3′), as described by Chen et al. [26]. Amplification products were extracted from 2% agarose gel, and purified using the AxyPrep DNA gel extraction kit (Axygen Biosciences, Union City, CA, USA) according to the manufacturer’s protocol. Purified amplicons were quantified fluorometrically using Quantus™ Fluorometer (Promega, Madison, WA, USA). High-quality amplicons were subjected to high-throughput sequencing by Majorbio Bio-Pharm Technology Co., Ltd., Shanghai, China. Raw sequencing reads were demultiplexed, trimmed, and merged using fastp (version 0.23.4) and FLASH (version 1.2.7), respectively. High-quality merged reads were clustered into operational taxonomic units (OTUs) at 97% sequence identity using UPARSE (version 11), with chimeric sequences identified and removed during clustering. Taxonomic assignment of representative OTU sequences was conducted using RDP Classifier (version 11.5) against the silva138/16s_bacteria database, with a confidence threshold of 0.7, as recommended by Wang et al. [27]. Sequences classified as chloroplast or mitochondrial origin were filtered out to eliminate host-derived contamination and primer bias artifacts. The resulting high-quality, taxonomy-annotated OTU table was used for all subsequent microbial community analyses. Sequencing depth (66,913 reads per sample) and Good’s coverage (99.83%) were calculated to evaluate sequencing completeness, and rarefaction analysis was performed to normalize samples to the same sequencing depth and eliminate the bias of uneven sequencing volume [28]. Alpha-diversity indices (Ace and Shannon) were calculated using MOTHUR software (version 1.30.2). The diagrams of Venn, bacterial community composition, principal co-ordinates analysis (PCoA) and heatmap were generated using the R software (version 3.3.1) [25].

### 2.8. Resistance to P. aeruginosa Infection Assay

Prior to the bacterial challenge, *P. aeruginosa* HX-1, originally isolated from the hepatopancreas of *E. sinensis* and subsequently identified phenotypically and molecularly [4], was employed as the challenge strain. *P. aeruginosa* HX-1 was inoculated into NB and incubated at 30 °C and 180 r/min for 24 h. Cells were harvested by centrifugation at 4000 r/min for 10 min at 4 °C, washed with sterile distilled water, and suspended in sterile distilled water to yield a uniform cell suspension [4]. The suspension was then uniformly sprayed onto the sterile commercial basal diet described in Section 2.3, thoroughly mixed, and air-dried under aseptic conditions at 25 °C [29]. The cell density in the feed was verified as 5.0 × 10^6^ CFU/g diet by plating serial dilutions on NA plates following 10-fold serial dilution in sterile distilled water. Ten crabs were randomly selected from each of the control and treatment aquaria immediately post the feeding trial, and transferred to challenge aquaria (78 cm × 52 cm × 48 cm) supplied with 120 L aerated tap water at 28 °C [4]. Three replicate challenge aquaria per group (10 crabs per aquarium) were orally challenged as recommended by Cao et al. [13] by being fed the 5.0 × 10^6^ CFU/g diet [13] of *P. aeruginosa*—supplemented diets twice daily (8:00 and 18:00) to apparent satiation, at 3% of the total body weight as described by Fu et al. [21]. Throughout the 7-day challenge period as recommended by Cao et al. [30], the test crabs were maintained under a 12 h dark: 12 h light photoperiod, uneaten food was removed from aquaria 2 h post-prandially, and 1/3 of the aquarium water was replaced daily with fresh aerated tap water [4]. Mortality was recorded daily, and cumulative survival rates were calculated over the 7-day observation period. All deceased crabs were promptly removed, and their hepatopancreas tissues were aseptically excised for re-isolation and molecular identification to confirm the challenge strain-attributed mortality. Relative percentage survival (RPS) was calculated using the formula proposed by Abdel-Latif et al. [31].

RPS (%) = (1 − mortality in experimental group/mortality in control group) × 100%

### 2.9. Statistical Analysis

All data are expressed as the mean ± standard deviation (SD). The enzymatic activity data were analyzed using non-parametric Kruskal–Wallis H test with a statistical confidence level of 95%. Microbiota community data were subjected to non-parametric multivariate analysis. Survival rates were analyzed using Kaplan–Meier method and log rank test in the SPSS 18.0 software (SPSS Inc., Chicago, IL, USA). Statistical difference is defined at *p* < 0.05.

## 3. Results

### 3.1. Effect of M. varians P2-4 Supplementation on Nonspecific Immunity of Crabs

The effect of *M. varians* P2-4 supplementation on nonspecific immune parameters in crabs is shown in Table 1. Neither molting nor mortality was observed in all experimental groups. Compared with the control, *M. varians* P2-4 supplementation at 6.0 × 10^6^ to 6.0 × 10^7^ CFU/g diet significantly increased plasma LZM activity in crabs by 9.09% (*p* < 0.05)~24.24% (*p* < 0.05). In contrast, plasma ACP and AKP activities were slightly elevated by 117.94% (*p* > 0.05)~226.91% (*p* > 0.05) and 34.51% (*p* > 0.05)~39.61% (*p* > 0.05), respectively. Similarly, across the same supplementation range, hepatopancreatic ACP and AKP activities were slightly raised by 18.46% (*p* > 0.05)~20.00% (*p* > 0.05) and 2.50% (*p* > 0.05)~5.00% (*p* > 0.05), respectively. Notably, in the test crabs fed diets containing 6.0 × 10^8^ CFU/g diet of *M. varians* P2-4, plasma ACP, AKP and LZM activities were significantly enhanced by 290.50% (*p* < 0.05), 56.98 (*p* < 0.05) and 43.94% (*p* < 0.05), respectively. Meanwhile, hepatopancreatic AKP activity was significantly raised by 7.50% (*p* < 0.05) while hepatopancreatic ACP activity was only increased by 26.15% (*p* > 0.05). Collectively, these findings demonstrate that dietary supplementation with *M. varians* P2-4 boosts nonspecific immune responses in *E. sinensis*.

### 3.2. Effect of M. varians P2-4 Supplementation on Antioxidant Capability in Crabs

The effect of *M. varians* P2-4 supplementation on antioxidant capability in crabs is presented in Table 2. Compared with the control, hepatopancreatic SOD and CAT activities were significantly elevated by 16.07% (*p* < 0.05)~29.46% (*p* < 0.05) and 2.00% (*p* < 0.05)~4.78% (*p* < 0.05), respectively, in the test crabs fed diets containing 6.0 × 10^6^ to 6.0 × 10^8^ CFU/g diet of *M. varians* P2-4. Similarly, across the same supplementation range, plasma SOD activity was significantly increased by 2.32% (*p* < 0.05)~17.16% (*p* < 0.05) while plasma CAT activity was slightly raised by 26.48% (*p* > 0.05)~44.66% (*p* > 0.05). Collectively, these findings revealed that dietary supplementation with *M. varians* P2-4 enhances antioxidant capability in *E. sinensis*.

### 3.3. Effect of M. varians P2-4 Supplementation on Intestinal Microbiota of Crabs

**Figure 1:**
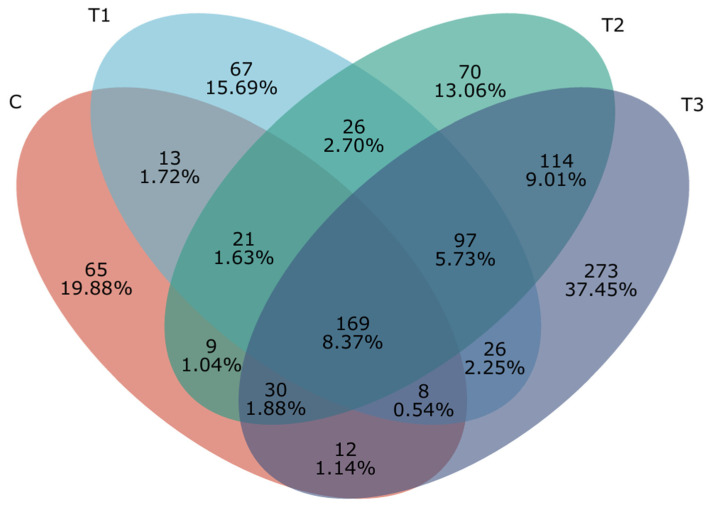
Venn diagram showing the distribution of OTUs presented in the intestine of crabs fed with *M. varians* P2-4-supplemented diets. The numbers indicate the correlated OTUs in the intestine of *M. varians* P2-4−fed crabs. C, 0 CFU/g diet; T1, 6.0 × 10^6^ CFU/g diet; T2, 6.0 × 10^7^ CFU/g diet; T3, 6.0 × 10^8^ CFU/g diet.

**Figure 2:**
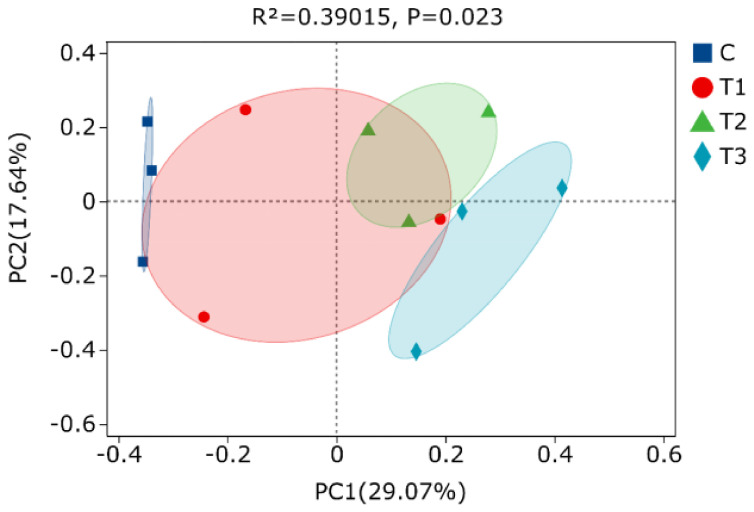
Principal coordinate analysis (PCoA) of intestinal bacterial communities in crabs fed with *M. varians* P2-4−supplemented diets, plotted using Bray–Curtis dissimilarity and ADONIS test. C, 0 CFU/g diet; T1, 6.0 × 10^6^ CFU/g diet; T2, 6.0 × 10^7^ CFU/g diet; T3, 6.0 × 10^8^ CFU/g diet.

**Figure 3:**
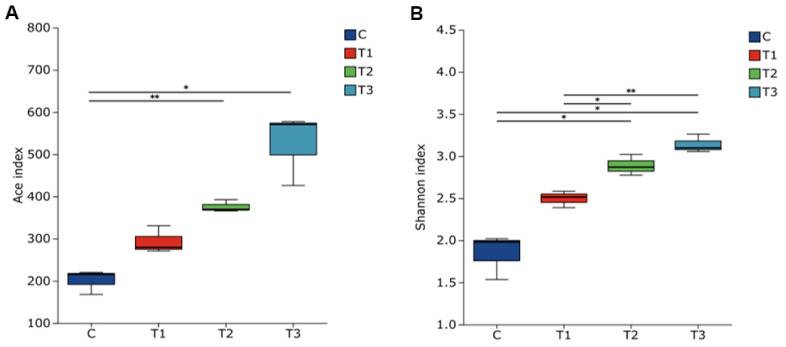
Alpha diversity of intestinal microbiota in crabs fed with *M. varians* P2-4−supplemented diets. (**A**). Richness of intestinal microbiota indicated by Ace index; (**B**). Diversity of intestinal microbiota indicated by Shannon index. C, 0 CFU/g diet; T1, 6.0 × 10^6^ CFU/g diet; T2, 6.0 × 10^7^ CFU/g diet; T3, 6.0 × 10^8^ CFU/g diet. Values are presented as mean ± SD (*n* = 3). Asterisks indicate statisti-cally significant difference (*, *p* < 0.05; **, *p* < 0.01).

**Figure 4:**
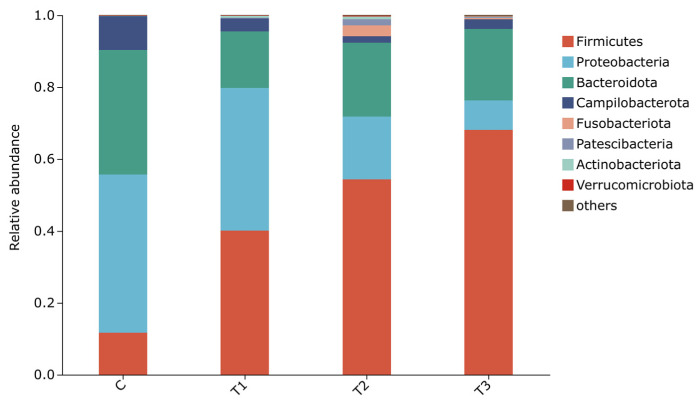
Composition of intestinal bacterial communities in crabs fed with *M. varians* P2-4−supplemented diets at the phylum level. C, 0 CFU/g diet; T1, 6.0 × 10^6^ CFU/g diet; T2, 6.0 × 10^7^ CFU/g diet; T3, 6.0 × 10^8^ CFU/g diet.

**Figure 5:**
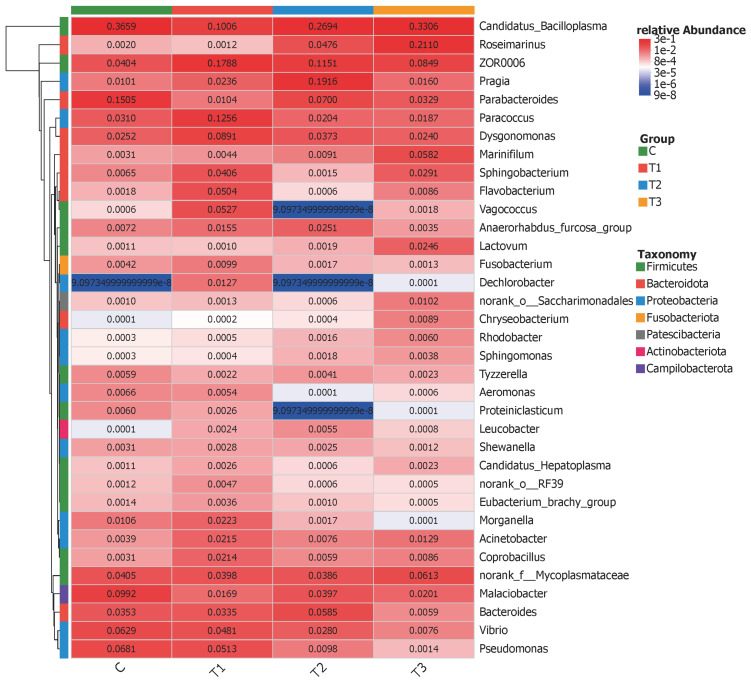
Heatmap analysis of intestinal bacterial communities in crabs fed with *M. varians* P2-4−supplemented diets at the genus level. C, 0 CFU/g diet; T1, 6.0 × 10^6^ CFU/g diet; T2, 6.0 × 10^7^ CFU/g diet; T3, 6.0 × 10^8^ CFU/g diet.

A total of 65, 67, 70, and 273 unique OTUs were detected in the intestinal microbiota of control crabs and those fed diets supplemented with 6.0 × 10^6^ to 6.0 × 10^8^ CFU/g diet of *M. varians* P2-4, respectively. Notably, 169 OTUs were shared between the control and *M. varians* P2-4-fed crabs (Figure 1), which mainly belonged to *Firmicutes* (44.22%), *Proteobacteria* (27.19%), *Bacteroidota* (23.42%), and *Campilobacterota* (4.69%). PCoA revealed distinct clustering patterns, indicating significant differences in intestinal microbial community composition between the control and *M. varians* P2-4-fed crabs (Figure 2). Compared with the control, 6.0 × 10^6^ CFU/g diet of *M. varians* P2-4 supplementation caused slight increases of 46.14% (*p* > 0.05) in Ace index and 35.33% (*p* > 0.05) in Shannon index. Notably, the Ace and Shannon indices were significantly elevated by 87.12% (*p* < 0.05)~161.17% (*p* < 0.05) and 57.07% (*p* < 0.05)~70.65% (*p* < 0.05), respectively, in crabs fed 6.0 × 10^7^ to 6.0 × 10^8^ CFU/g diet of *M. varians* P2-4-supplemented diets (Figure 3), suggesting enhanced microbial richness and diversity. At the phylum level, compared with the control, the abundance of *Firmicutes* was significantly increased by 242.36% (*p* < 0.05)~481.21% (*p* < 0.05) in 6.0 × 10^6^ to 6.0 × 10^8^ CFU/g diet of *M. varians* P2-4-fed crabs, while the abundance of *Bacteroidota* decreased markedly by 40.71% (*p* < 0.05)~54.93% (*p* < 0.05). Furthermore, the abundance of *Proteobacteria* was significantly reduced by 60.45% (*p* < 0.05)~81.23% (*p* < 0.05) in crabs fed diets containing 6.0 × 10^7^ to 6.0 × 10^8^ CFU/g diet of *M. varians* P2-4, and only slightly decreased by 9.61% (*p* > 0.05) at 6.0 ×10^6^ CFU/g diet (Figure 4). At the genus level, although no live *Massilia* were detected in the intestine, the abundance of *Rhodobacter* increased significantly by 66.67% (*p* < 0.05)~1900.00% (*p* < 0.05) in 6.0 × 10^6^ to 6.0 × 10^8^ CFU/g diets of *M. varians* P2-4-fed crabs compared to the control. Meanwhile the abundance of *Aeromonas* decreased markedly by 18.18% (*p* < 0.05)~98.48% (*p* < 0.05) (Figure 5). Collectively, these findings demonstrate that dietary supplementation with *M. varians* P2-4 improves both composition and diversity of intestinal microbiota in *E. sinensis*.

### 3.4. Effect of M. varians P2-4 Supplementation on Resistance of Crabs to P. aeruginosa Infection

**Figure 6:**
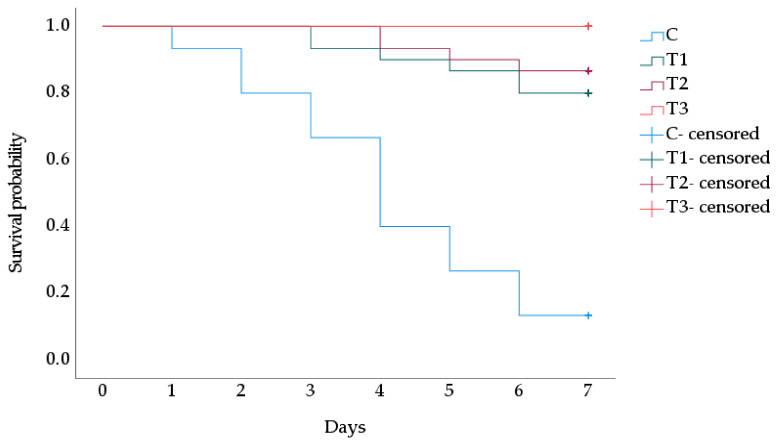
Effect of *M. varians* P2-4 supplementation on resistance of crabs to *P. aeruginosa* infection. C, 0 CFU/g diet; T1, 6.0 × 10^6^ CFU/g diet; T2, 6.0 × 10^7^ CFU/g diet; T3, 6.0 × 10^8^ CFU/g diet.

The effect of *M. varians* P2-4 supplementation on resistance of crabs to *P. aeruginosa* infection is shown in Figure 6. In the control group, acute mortality was observed following challenge with *P. aeruginosa*, with cumulative mortality reaching 86.67% by day 6. In contrast, crabs fed diets supplemented with 6.0 × 10^6^ to 6.0 × 10^8^ CFU/g diet of *M. varians* P2-4 exhibited significantly higher survival rates of 80.00%, 86.67%, and 100.00%, corresponding to survival rates significantly increased by 66.67% (*p* < 0.05), 73.34% (*p* < 0.05) and 86.67% (*p* < 0.05) as compared to the control. Consequently, the 7-day RPS of *M. varians* P2-4 at 6.0 × 10^6^ to 6.0 × 10^8^ CFU/g diet ranged from 76.9% to 100.0% following *P. aeruginosa* challenge. The challenge strain was successfully re-isolated from the deceased crabs and confirmed via phenotypic and molecular identification (Table S1, Figure S1). Collectively, these findings demonstrate that dietary *M. varians* significantly enhances resistance to *P. aeruginosa* infection in *E. sinensis*.

## 4. Discussion

To date, *P. aeruginosa* infection in *E. sinensis* can be managed mostly through the use of antibiotics [32,33]. However, such antibiotic-based interventions are economically unsustainable for aquaculturists and pose significant risks to environmental integrity and public health [34]. In response, several alternative strategies have recently emerged for controlling pathogenic *P. aeruginosa* in aquaculture, including the use of *Melaleuca alternifolia* essential oil, Yucca extract, *Chaetomorpha linum* extract, grape pomace flour, bacteriophages, *Bacillus coagulans*, *Bdellovibrio* powder, *Padina boergesenii*, and levamisole [4,35,36,37,38,39,40,41,42]. Despite this, evidence supporting the use of *M. varians* strains as a dietary intervention to prevent bacterial infection in crabs remains entirely lacking. In this study, we systematically evaluated the modulatory effects of *M. varians* P2-4 on nonspecific immunity, antioxidant capability, intestinal microbiota and resistance to *P. aeruginosa* infection in *E. sinensis*. Notably, *M. varians* P2-4 at 6.0 × 10^8^ CFU/g diet presented 16.67% higher RPS against *P. aeruginosa* infection in *E. sinensis* than *Bdellovibrio* powder at 15 g/kg diet [4], suggesting *M. varians* P2-4 as a promising agent against *P. aeruginosa* infection in *E. sinensis*. However, the current study has several inherent limitations that should be acknowledged, such as limited mechanistic evidence, absence of histopathology, lack of direct colonization and growth performance data, and uncertainty regarding the specific mode of protection.

In crustaceans, the innate immune system provides a critical first line of defense against invading pathogens through rapid nonspecific immune responses [43]. ACP, AKP and LZM are well-established enzymatic markers of innate immunity and play indispensable roles in pathogen recognition, microbial clearance, and immune homeostasis in crustaceans [44,45,46]. Elevated activities of these enzymes have been consistently associated with enhanced disease resistance in *E. sinensis* [47,48]. In this study, dietary supplementation with *M. varians* P2-4 significantly increased plasma ACP, AKP and LZM activities as well as hepatopancreatic ACP and AKP activities, collectively indicating a robust activation of the innate immune response in *E. sinensis*. It is speculated that such immunomodulatory effects might be related to changes in gene expression [49].

Crustaceans rely on free radicals during innate immune defense to exert antimicrobial function and defend themselves against invading pathogens [50]. However, excessive free radicals can induce significant oxidative stress, leading to cytotoxic damage such as DNA damage, lipid peroxidation and protein oxidation [51,52]. SOD and CAT are pivotal enzymatic scavengers of excessive free radicals during immune response, and constitute essential components of the first line of cellular defense against pathogen-induced oxidative injury [51,52]. Elevated activities of these enzymes have been consistently correlated with enhanced resistance to various pathogens in crustaceans [53]. In this study, dietary supplementation with *M. varians* P2-4 significantly increased SOD and CAT activities in both plasma and hepatopancreas of crabs, suggesting that *M. varians* P2-4 confers protection against oxidative damage that occurs during the pathogen challenge. The observed effects might be associated with the regulation of these antioxidant enzymes’ gene expression [49].

Dietary supplementation with probiotic microorganisms has been shown to enhance intestinal microbial diversity in *E. sinensis* [13,47]. Likewise, the present study demonstrated that dietary supplementation with *M. varians* P2-4 significantly increased intestinal microbial diversity, as indicated by the increased alpha-diversity indices combined with PCoA, collectively indicating a more stable intestinal microbiota community structure in *M. varians* P2-4-fed *E. sinensis* compared to control. Such enhanced microbial stability significantly contributes to inhibiting the invasion of pathogenic bacteria and enhancing host non-specific immunity [54]. Furthermore, *M. varians* P2-4 supplementation markedly increased the abundance of *Firmicutes* and *Rhodobacter*. These taxa have been reported to participate in the immunity and disease resistance of *E. sinensis* via improving immune-related enzyme activities and intestinal metabolism [13,47,48,55]. Similar findings have also been reported in *E. sinensis* after intervention with probiotics such as *B. licheniformis* and *R. sphaeroides* [47,48]. In addition, *M. varians* P2-4 supplementation markedly reduced the abundance of *Aeromonas*, which has been documented to correlate with hepatopancreatic necrosis syndrome, enteritis and other severe infections in *E. sinensis* [56,57,58]. This underscores the capacity of *M. varians* P2-4 to promote intestinal microeubiosis and host health. The underlying mechanism may mainly rely on direct predation in the intestine [8], and might also involve secretion of antimicrobial compounds that potentially inhibit pathogen colonization [9,59].

Bacterial disease outbreaks have become the most preventive factor in crab production under intensive culture [48]. *P. aeruginosa*, as an emerging bacterial pathogen of *E. sinensis* [4], is ubiquitous in natural settings such as soil and water [60]. This bacterium possesses numerous virulence factors that mediate bacterial adhesion, biofilm formation, and the secretion of exoenzymes and exotoxins [4]. It also exhibits multiple drug resistance to aminoglycosides, fluoroquinolones, and penicillins commonly used in aquaculture [4]. Therefore, *P. aeruginosa* is a major threat to *E. sinensis*, and development of defense strategies to survive its infection is urgently required to avoid the spread of this threat [4]. In the present study, dietary supplementation with *M. varians* P2-4 at 6.0 × 10^6^ to 6.0 × 10^8^ CFU/g diet increased the survival rate of *E. sinensis* in the challenge test to above 80.00%. The observed protective effect is speculated to be associated with the modulation of host immune response and intestinal microecology [61]. However, given the absence of higher and lower dose groups in the present study, we cannot confirm whether the maximum tested dose reached a plateau effect, nor accurately determine the exact minimum effective dose. Further multi-gradient dose verification is required in the future to elaborate the specific pattern of this dose–response relationship.

## 5. Conclusions

Dietary supplementation with *M. varians* P2-4 effectively improved antioxidant and immune enzyme status, reshaped intestinal microbial community structure and diversity, and enhanced survival of *E. sinensis* to *P. aeruginosa* infection in *E. sinensis*. These findings establish *M. varians* P2-4 supplementation as a promising biocontrol strategy for enhancing disease resistance in *E. sinensis*.

## Figures and Tables

**Table 1:** Effect of *M. varians* P2-4 supplementation on nonspecific immune parameters in *E. sinensis*.

Cell Density (CFU/g)	Plasma Immune Enzymes (U/mL)			Hepatopancreatic Immune Enzymes (U/mg Protein)	
	ACP	AKP	LZM	ACP	AKP
0	3.79 ± 0.60 ^a^	8.81 ± 0.52 ^a^	5.28 ± 0.16 ^a^	1.30 ± 0.05 ^a^	0.40 ± 0.01 ^a^
6.0 × 10^6^	8.26 ± 3.32 ^ab^	11.85 ± 0.19 ^ab^	5.76 ± 0.11 ^b^	1.54 ± 0.21 ^a^	0.41 ± 0.02 ^ab^
6.0 × 10^7^	12.39 ± 2.38 ^ab^	12.30 ± 0.50 ^ab^	6.56 ± 0.58 ^c^	1.56 ± 0.09 ^a^	0.42 ± 0.01 ^ab^
6.0 × 10^8^	14.80 ± 2.06 ^b^	13.83 ± 0.32 ^b^	7.60 ± 0.10 ^d^	1.64 ± 0.23 ^a^	0.43 ± 0.01 ^b^

Values are presented as mean ± SD (*n* = 3). Data represented by different superscript letters in the same column indicate statistically significant difference (*p* < 0.05). ACP, acid phosphatase; AKP, alkaline phosphatase; LZM, lysozyme.

**Table 2:** Effect of *M. varians* P2-4 supplementation on antioxidant parameters in *E. sinensis*.

Cell Density (CFU/g)	Plasma Antioxidant Enzymes (U/mL)		Hepatopancreatic Antioxidant Enzymes (U/mg Protein)	
	SOD	CAT	SOD	CAT
0	42.20 ± 0.19 ^a^	2.53 ± 0.81 ^a^	1.12 ± 0.04 ^a^	44.58 ± 0.12 ^a^
6.0 × 10^6^	43.18 ± 0.14 ^b^	3.20 ± 0.12 ^a^	1.30 ± 0.03 ^b^	45.47 ± 0.13 ^b^
6.0 × 10^7^	43.70 ± 0.30 ^c^	3.62 ± 0.43 ^a^	1.34 ± 0.02 ^b^	45.82 ± 0.16 ^c^
6.0 × 10^8^	49.44 ± 0.19 ^d^	3.66 ± 0.23 ^a^	1.45 ± 0.01 ^c^	46.71 ± 0.09 ^d^

Values are presented as mean ± SD (*n* = 3). Data represented by different superscript letters in the same column indicate statistically significant difference (*p* < 0.05). SOD, superoxide dismutase; CAT, catalase.

## Data Availability

The data that support the findings of this study are available from the corresponding author upon reasonable request.

## Supplementary Materials

The following supporting information can be downloaded at https://www.mdpi.com/article/10.3390/biology15120908/s1, Table S1: The phenotypic features of the reisolate from the experimentally deceased crabs. Figure S1: The 16S rRNA phylogenetic tree constructed using neighbor-joining method for the reisolate from the experimentally deceased crabs and 11 known bacteria.

## References

[1] Song Y., Wu M., Cheng Y., Niu C., Yu X., Pang Y., Yang X. (2024). The protective effects of microbe derived antioxidants on digestive tissue morphology, functions, and intestinal microbiota diversity of *Eriocheir sinensis* exposed to glyphosate. Aquac. Nutr..

[2] Wang A., Ma X., Xu J., Lu W. (2019). Methane and nitrous oxide emissions in rice-crab culture systems of northeast China. Aquac. Fish..

[3] Wang D., Gao H. (2024). China Fishery Statistical Yearbook.

[4] He L., Yang Y., Liu Y., Liu M., Cao H., Gai C., Ye W. (2026). *Pseudomonas aeruginosa* in Chinese mitten crab *Eriocheir sinensis*: Identification, pathogenic potential and control with *Bdellovibrio* powder. J. Invertebr. Pathol..

[5] Amirhosseini K., Alizadeh M., Azarbad H. (2025). Harnessing the ecological and genomic adaptability of the bacterial genus *Massilia* for environmental and industrial applications. Microb. Biotechnol..

[6] Lindquist D., Murrill D., Burran W.P., Winans G., Janda J.M., Probert W. (2003). Characteristics of *Massilia timonae* and *Massilia timonae*-like isolates from human patients, with an emended description of the species. J. Clin. Microbiol..

[7] Yang E., Cui D., Wang W. (2019). Rsearch progress on the genus *Massilia*. Microbiol. China.

[8] An J., Cao H., Yang Y., He L., Liu Y. (2025). A Predatory Strain of *Massilia varians* and Its Application.

[9] Sedláček I., Holochová P., Sedlář K., Staňková E., Šedo O., Kralova S., Umair M., Koublová V., Švec P. (2025). Two new psychrotolerant *Massilia* species inhibit plant pathogens *Clavibacter* and *Curtobacterium*. Sci. Rep..

[10] Liu Y., Yang Y., Liu M., Chen S., Cao H., Gai C., Ye W. (2026). *Massilia varians* P2-4, A potential biocontrol agent against pathogenic *Pseudomonas aeruginosa* in *Eriocheir sinensis*. bioRxiv.

[11] Huang Q., Wang R., Ding Q., Liao F., Zhu L., Huang M., Li J., Zeng J., Shen Q., Wang M. (2025). Low-nitrogen input enriches *Massilia* bacteria in the phyllosphere to improve blast resistance in rice. New Phytol..

[12] Amankwah F.K.D., Gbedema S.Y., Boakye Y.D., Bayor M.T., Boamah V.E. (2022). Antimicrobial potential of extract from a *Pseudomonas aeruginosa* isolate. Scientifica.

[13] Cao H., Zhang S., An J., Diao J., Xu L., Gai C. (2022). *Rhodobacter azotoformans* supplementation improves defense ability of Chinese mitten crab *Eriocheir sinensis* against citrobacteriosis. Fish Shellfish Immunol..

[14] Kumar N.R., Raman R.P., Jadhao S.B., Brahmchari R.K., Kumar K., Dash G. (2013). Effect of dietary supplementation of *Bacillus licheniformis* on gut microbiota, growth and immune response in giant freshwater prawn, *Macrobrachium rosenbergii* (de Man, 1879). Aquac. Int..

[15] Feng Y., Huang X., Wang K., Chen H., Wang J. (2020). Evaluation of the leading physical indicators of hepatopancreatic necrosisdisease of Chinese mitten crab (*Eriocheir sinensis*). J. Fish. China.

[16] Yang Z., Zhang Y., Hu K., Liu L., Cai H., Zhang F., Yang X. (2018). Etiological and histopathological study on hepatopancreatic necrosis syndrome in *Eriocheir sinensis*. Acta Hydrobiol. Sin..

[17] Ding Z., Yao Y., Zhang F., Wan J., Sun M., Liu H., Zhou G., Tang J., Pan J., Xue H. (2015). The first detection of white spot syndrome virus in naturally infected cultured Chinese mitten crabs, *Eriocheir sinensis* in China. J. Virol. Methods.

[18] Xiang A., Chen J., Sun J., Weng C., Cheng Y., Yang X. (2025). Dietary trehalose activates autophagy potentially via MAPK signaling pathway to alleviate oxidative stress induced by air exposure stress in Chinese mitten crab (*Eriocheir sinensis*). Aquac. Nutr..

[19] Wang X., Huang Z., Wang C., Qi C., Gu Z., Li E., Qin J.G., Chen L. (2020). A comparative study on growth and metabolism of *Eriocheir sinensis* juveniles under chronically low and high pH stress. Front. Physiol..

[20] Dai F., Song L., Gao J., Tai X., Chu L., Zhuang H., Shao N., Hu J., Nei Z., Wang Y. (2020). Effect of stocking density on mortality rate, physiological status and nutrient contents of Chinese mitten crab *Eriocheir sinensis* during overwintering cultivation. Aquac. Rep..

[21] Fu L., Zhou G., Pan J., Li Y., Lu Q., Zhou J., Li X. (2017). Effects of *Astragalus polysaccharides* on antioxidant abilities and non-specific immune responses of Chinese mitten crab, *Eriocheir sinensis*. Aquac. Int..

[22] Li Y., Chai X., Wu H., Jing W., Wang L. (2013). The response of metallothionein and malondialdehyde after exclusive and combined Cd/Zn exposure in the crab *Sinopotamon henanense*. PLoS ONE.

[23] Xu H., Dou J., Wu Q., Ye Y., Song C., Mu C., Wang C., Ren Z., Shi C. (2022). Investigation of the light intensity effect on growth, molting, hemolymph lipid, and antioxidant capacity of juvenile swimming crab *Portunus trituberculatus*. Front. Mar. Sci..

[24] Mager S., Oomen M.H., Morente M.M., Ratcliffe C., Knox K., Kerr D.J., Pezzella F., Riegman P.H. (2007). Standard operating procedure for the collection of fresh frozen tissue samples. Eur. J. Cancer.

[25] Hou M., Pang Y., Niu C., Zhang D., Zhang Y., Liu Z., Song Y., Shi A., Chen Q., Zhang J. (2022). Effects of dietary L-TRP on immunity, antioxidant capacity and intestinal microbiota of the Chinese mitten crab (*Eriocheir sinensis*) in pond culture. Metabolites.

[26] Chen X., Chen H., Liu Q., Ni K., Ding R., Wang J., Wang C. (2020). High plasticity of the gut microbiome and muscle metabolome of Chinese mitten crab (*Eriocheir sinensis*) in diverse environments. J. Microbiol. Biotechnol..

[27] Wang Q., Garrity G.M., Tiedje J.M., Cole J.R. (2007). Naive Bayesian classifier for rapid assignment of rRNA sequences into the new bacterial taxonomy. Appl. Environ. Microbiol..

[28] Neuendorf E., Gajer P., Bowlin A.K., Marques P.X., Ma B., Yang H., Fu L., Humphrys M.S., Forney L.J., Myers G.S. (2015). *Chlamydia caviae* infection alters abundance but not composition of the guinea pig vaginal microbiota. Pathog. Dis..

[29] Chauhan A., Singh R. (2019). Isolation and evaluation of putative probiotic strains from different teleost to prevent *Pseudomonas aeruginosa* infection in *Cyprinus carpio*. Aquac. Res..

[30] Cao H., Zhang S., Zheng X., Xu L., Diao J., Wang Y., Gai C., Ye H. (2023). Safety assessment of *Rhodobacter azotoformans* SY5 for potential application in Chinese mitten crab *Eriocheir sinensis*. Benef. Microbes.

[31] Abdel-Latif H.M., Abdel-Tawwab M., Khafaga A.F., Dawood M.A. (2020). Dietary origanum essential oil improved antioxidative status, immune-related genes, and resistance of common carp (*Cyprinus carpio* L.) to *Aeromonas hydrophila* infection. Fish Shellfish Immunol..

[32] Kholil M.I., Hossain M.M.M., Neowajh M.S., Islam M.S., Kabir M. (2015). Comparative efficiency of some commercial antibiotics against *Pseudomonas* infection in fish. Int. J. Fish. Aquat. Stud..

[33] Ali N.G., Ali T.E.-S., Aboyadak I.M., Elbakry M.A. (2021). Controlling *Pseudomonas aeruginosa* infection in *Oreochromis niloticus* spawners by cefotaxime sodium. Aquaculture.

[34] Founou L.L., Founou R.C., Essack S.Y. (2016). Antibiotic resistance in the food chain: A developing country-perspective. Front. Microbiol..

[35] Souza C.F., Baldissera M.D., Santos R.C., Raffin R.P., Baldisserotto B. (2017). Nanotechnology improves the therapeutic efficacy of *Melaleuca alternifolia* essential oil in experimentally infected *Rhamdia quelen* with *Pseudomonas aeruginosa*. Aquaculture.

[36] El-Keredy M.A., Naena N.A. (2020). Yucca plant as treatment for *Pseudomonas aeruginosa* infection in *Nile tilapia* farms with emphasis on its effect on growth performance. Alex. J. Vet. Sci..

[37] Thanigaivel S., Thomas J., Vickram A., Gulothungan G., Nanmaran R., Jenila Rani D. (2023). Antioxidant and antibacterial efficacy of *Chaetomorpha linum* and its toxicological evaluation for the prophylactic treatment against *Pseudomonas aeruginosa* infection in *Labeo rohita*. J. Appl. Aquac..

[38] Baldissera M.D., Souza C.F., Descovi S.N., Verdi C.M., Zeppenfeld C.C., de Lima Silva L., Gindri A.L., Cunha M.A., Santos R.C., Baldisserotto B. (2019). Effects of dietary grape pomace flour on the purinergic signaling and inflammatory response of grass carp experimentally infected with *Pseudomonas aeruginosa*. Aquaculture.

[39] Richards G.P. (2014). Bacteriophage remediation of bacterial pathogens in aquaculture: A review of the technology. Bacteriophage.

[40] Ji T., Cao Y., Cao Q., Zhang Y., Yang H. (2022). The antagonistic effect and protective efficacy of gram-positive probiotics *Bacillus coagulans* to newly identified pathogens *Pseudomonas aeruginosa* in crucian carp *Carassius auratus gibelio*. Aquac. Rep..

[41] Ragunath C., Ramasubramanian V. (2022). Dietary effect of Padina boergesenii on growth, immune response, and disease resistance against *Pseudomonas aeruginosa* in *Cirrhinus mrigala*. Appl. Biochem. Biotechnol..

[42] El-Gammal G.A., El-Gamal A.M., Rashed M., Kassab A.S., Saif A.S., Fadl S.E. (2025). An experimental study of levamisole incorporated diet on fish health and resistance against *Pseudomonas aeruginosa* isolated from *Oreochromas niloticus*. Sci. Rep..

[43] He Y., Yuan X., Li J., Tian X., He Z., Zeng C., Xie Y., Liu L., Deng S., Wang D. (2025). *Citrobacter freundii* caused head ulcer disease and immune response in juvenile *Procambarus clarkii*. Fishes.

[44] Wang C., Wang R., Yang H., Wang Y., Zhang Z. (2022). Gene cloning and transcriptional regulation of the alkaline and acid phosphatase genes in *Scylla paramamosain*. Gene.

[45] Li W.J., Li T., Qiao H.L., Wang C., Jin Z.Y., Zhang H.W., Xia X.-H., Zhang X.W. (2025). Immune function of a novel lysozyme in *Litopenaeus vannamei* and its application as a feed supplement. Int. J. Biol. Macromol..

[46] Chen J., Du Y., Zhang M., Wang J., Ming J., Shao X., Wang A., Tian H., Zhang W., Xia S. (2025). Effects of melatonin on the growth and diurnal variation of non-specific immunity, antioxidant capacity, digestive enzyme activity, and circadian clock-related gene expression in crayfish (*Procambarus clarkii*). Fishes.

[47] Cao H., Zheng X., Teng C., Xu L., Wang Y., Gai C., Ye H. (2024). *Rhodobacter sphaeroides* supplementation improves defense ability of Chinese mitten crab *Eriocheir sinensis* against *Shewanella putrefaciens* infection via intestinal flora and metabolism regulation. J. Invertebr. Pathol..

[48] Cao H., Huang X., Gu Y., Zheng X., Xu L., Gai C. (2022). Protective effects of *Bacillus licheniformis* against *Citrobacter freundii* infection in Chinese mitten crab *Eriocheir sinensis*. J. Invertebr. Pathol..

[49] Ji P.F., Yao C.L., Wang Z.Y. (2009). Immune response and gene expression in shrimp (*Litopenaeus vannamei*) hemocytes and hepatopancreas against some pathogen-associated molecular patterns. Fish Shellfish Immunol..

[50] Rattanachai A., Hirono I., Ohira T., Takahashi Y., Aoki T. (2004). Cloning of kuruma prawn *Marsupenaeus japonicus* crustin-like peptide cDNA and analysis of its expression. Fish. Sci..

[51] Ren X., Lv J., Gao B., Li J., Liu P. (2017). Immune response and antioxidant status of *Portunus trituberculatus* inoculated with pathogens. Fish Shellfish Immunol..

[52] He Q., Feng W., Chen X., Xu Y., Zhou J., Li J., Xu P., Tang Y. (2024). H_2_O_2_-induced oxidative stress responses in *Eriocheir sinensis*: Antioxidant defense and immune gene expression dynamics. Antioxidants.

[53] Larbi Ayisi C., Zhao J., Wu J.-W. (2018). Replacement of fish oil with palm oil: Effects on growth performance, innate immune response, antioxidant capacity and disease resistance in Nile tilapia (*Oreochromis niloticus*). PLoS ONE.

[54] Mazón-Suástegui J.M., Salas-Leiva J.S., Medina-Marrero R., Medina-García R., García-Bernal M. (2020). Effect of *Streptomyces* probiotics on the gut microbiota of *Litopenaeus vannamei* challenged with *Vibrio parahaemolyticus*. MicrobiologyOpen.

[55] Zhou R., Liu J., Shi X., Fu C., Jiang Y., Zhang R., Wu Y., Yang C. (2022). Garlic powder supplementation improves growth, nonspecific immunity, antioxidant capacity, and intestinal flora of Chinese mitten crabs (*Eriocheir sinensis*). Aquac. Nutr..

[56] Zheng N., Wang N., Wang Z., Abdallah G., Zhang B., Wang S., Yao Q., Chen Y., Wang Q., Zhang D. (2022). Effect of infection with *Aeromonas hydrophila* on antioxidant capacity, inflammation response, and apoptosis proteins in Chinese mitten crab (*Eriocheir sinensis*). Comp. Biochem. Physiol. C Toxicol. Pharmacol..

[57] Zhou H., Huang X., An J., Cao H., Yang X. (2019). Isolation, identification and antibiotic susceptbility of pathogenic *Aeromonas veronii* in Chinese mitten crab *Eriocheir sinensis* and its histopathological observations. J. South. Agric..

[58] Cao H., Wen L., Bai J., Zhang F., He S. (2015). Isolation, identification and antimicrobial susceptibility of *Aeromonas punctata* from *Eriocheir sinensis*. Prog. Vet. Med..

[59] Sedláček I., Holochová P., Busse H.-J., Koublová V., Králová S., Švec P., Sobotka R., Staňková E., Pilný J., Šedo O. (2022). Characterisation of waterborne psychrophilic *Massilia* isolates with violacein production and description of *Massilia antarctica* sp. nov. Microorganisms.

[60] Crone S., Vives-Flórez M., Kvich L., Saunders A.M., Malone M., Nicolaisen M.H., Martínez-García E., Rojas-Acosta C., Catalina Gomez-Puerto M., Calum H. (2020). The environmental occurrence of *Pseudomonas aeruginosa*. APMIS.

[61] Ayiku S., Shen J., Tan B., Dong X., Liu H. (2020). Effects of dietary yeast culture on shrimp growth, immune response, intestinal health and disease resistance against *Vibrio harveyi*. Fish Shellfish Immunol..

