# Supplementary material for: Massilia varians P2-4 Supplementation Enhances Immunity, Antioxidant Capability, Intestinal Microbiota Diversity, and Disease Resistance Against Pseudomonas aeruginosa Infection in Chinese Mitten Crab Eriocheir sinensis
Source: Biology (Basel). 2026 Jun 10;15(12):908. doi: 10.3390/biology15120908 (PMC13295881; doi:10.3390/biology15120908)
Supplement: Supplementary file 1 [file biology-15-00908-s001.zip › biology-4322120-supplementary.pdf]

Table S1. The phenotypic features of the reisolate from the experimentally deceased crabs.

**Test**	**Reaction**	
	**the reisolate**	**HX-1 [4]**
Potassium nitrate	+	+
L-tryptophane	-	-
D-glucose	-	-
L-arginine	+	+
Urease	+	+
Aesculin	-	-
Gelatin	+	+
β-galactopyranoside	-	-
Glucose assimilation	+	+
D-mannose	-	-
Mannitol	+	+
N-Acetylglucosamine	+	+
Maltose	-	-
Potassium gluconate	+	+
Decanoic acid	+	+
Adipic acid	+	+
Malic acid	+	+
Trisodium citrate	+	+
Phenylacetate	-	-

The phenotypic featues were analyzed using the API 20NE system (BioMerieux, Lyon, France). +, positive reaction; -, negative reaction.

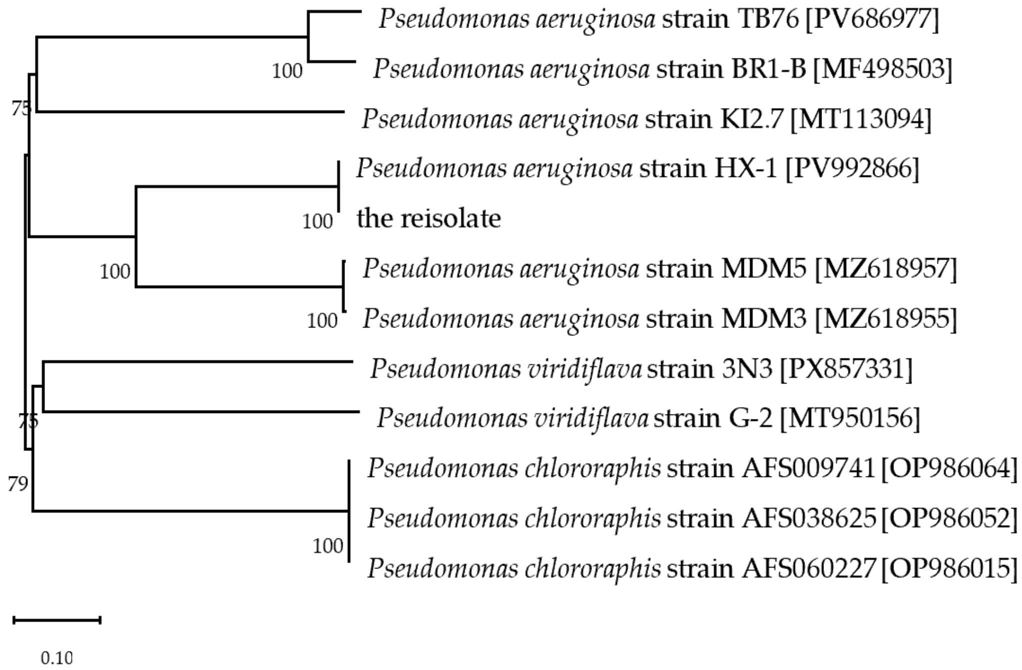

Figure S1. The 16S rRNA phylogenetic tree constructed using neighbor-joining method for the reisolate from the experimentally deceased crabs and 11 known bacteria. The bootstrap values (%) are shown beside the clades, accession numbers are indicated beside the name of strains, and scale bars represent distance values.
